# Tract integrity in amyotrophic lateral sclerosis: 6–month evaluation using MR diffusion tensor imaging

**DOI:** 10.1186/s12880-019-0319-3

**Published:** 2019-02-22

**Authors:** Ashwag R. Alruwaili, Kerstin Pannek, Robert D. Henderson, Marcus Gray, Nyoman D. Kurniawan, Pamela A. McCombe

**Affiliations:** 1 Faculty of Medicine, The University of Queensland, Australia and King Saud University, Brisbane, Australia; 2 The Australian e-Health Research Centre, CSIRO, Brisbane, Australia; 3 Department of Neurology, Faculty of Medicine, Royal Brisbane and Women’s Hospital and The University of Queensland, Brisbane, Australia; 4 Centre for Advanced Imaging, The University of Queensland, Brisbane, Australia; 5 Faculty of Medicine, UQ Centre for Clinical Research, Royal Brisbane and Women’s Hospital, The University of Queensland, Herston, QLD 4029 Australia

**Keywords:** Amyotrophic lateral sclerosis, Motor neuron disease, Diffusion tensor imaging, Voxel based morphometry, Tract-based spatial statistics, Cognitive impairment

## Abstract

**Background:**

This study was performed to assess changes in diffusion tensor imaging (DTI) over time in patients with amyotrophic lateral sclerosis (ALS).

**Methods:**

We performed DTI in 23 ALS patients who had two magnetic resonance imaging (MRI) scans at 6 month intervals and to correlate results with clinical features. The revised ALS functional rating scale (ALSFRS–R) was administered at each clinical visit. Data analysis included voxel–based white matter tract–based spatial statistics (TBSS) and atlas–based region–of–interest (ROI) analysis of fractional anisotropy (FA) and mean diffusivity (MD).

**Results:**

With TBSS, there were no significant changes between the two scans. The average change in FA and MD in the ROIs over 6 months was small and not significant after allowing for multiple comparisons. After allowing for multiple comparisons, there was no significant correlation of FA or MD with ALSFRS–R.

**Conclusion:**

This study shows that there is little evidence of progressive changes in DTI over time in ALS. This could be because white matter is already substantially damaged by the time of onset of symptoms of ALS.

## Background

Amyotrophic lateral sclerosis (ALS) is a neurodegenerative disease defined by loss of upper and lower motor neurons. However, extra–motor dysfunction can also be found in ALS [1] with cognitive impairment being prominent [2]. Magnetic resonance imaging (MRI) studies of ALS have reported pathological changes in white matter (WM), using diffusion tensor imaging (DTI), and in gray matter (GM) [3, 4]. There are changes in major white matter tracts in ALS [5], and changes in gray and white matter are more severe in patients with cognitive impairment [6].

For clinical monitoring and for use in clinical trials there is a need for a biomarker that is related to disease pathology [7]. Imaging biomarkers have the advantage of being non-invasive. DTI provides a measure of white matter changes. In ALS, cross–sectional studies have focused on abnormalities in the corticospinal tract (CST) [8–10], where it is widely accepted that there is reduction in fractional anisotropy (FA), although some studies have reported the involvement of association tracts and subcortical structures [11, 12]. Changes in mean diffusivity (MD) are less frequently studied, although in our previous study we found that MD shows changes in ALS [6]. It might be expected that serial DTI studies could be useful as a measure of the rate of progression of disease and could also demonstrate the spread of pathology to different regions [13].

There have been some longitudinal studies of DTI in ALS [14–17]. These have given inconsistent findings. Using a region of interest approach, some have reported the progression of ALS by demonstrating decreasing FA over time along the CST [14, 15], while another found that FA does not change on later scans [16]. There have been some recent studies suggesting changes in axial diffusivity (AD) in the spinal cord over time [18] and radial diffusivity (RD) over time [19]. A study using ^1^H magnetic resonance spectroscopy gave negative findings [17]. To obtain further value from MRI biomarkers, there is also a need for correlation of MRI findings with clinical status.

This study explores the changes in WM of motor and extra-motor pathways over time using tract-based spatial statistics (TBSS) and region-of-interest (ROI) analysis in 23 patients who had two MRI scans, 6 months apart, analyzing FA and MD. The primary hypothesis was that the later scan would show greater WM damage than the first scan and the secondary hypothesis was that changes in WM would correlate with changes in disease severity.

## Methods

### Participants

Patients with ALS were recruited from the multidisciplinary Motor Neuron Disease clinic at the Royal Brisbane Women Hospital (RBWH). All patients fulfilled the criteria for definite ALS according to revised El Escorial criteria [20]. The patients were classified into the phenotypes described by Chio et al. [21]. All participants in the present study were participants in a previous cross-sectional MRI study of 30 subjects with ALS [6]. The revised ALS functional rating scale (ALSFRS-R) was administered at each clinical visit [22]. All ALS subjects also received cognitive and behavioral testing, using the Addenbrooke’s cognitive examination III (ACE-III) [23, 24] and the Frontal Assessment battery (FAB) [25] on the day of the first MRI scan. The study was approved by the RBWH Human Research Ethics Committee (HREC 2008/98) and all patients provided written informed consent. All activities were conducted in accordance with relevant guidelines.

### Image acquisition

MRI scans were performed at RBWH using a 3 T Siemens Tim Trio (Siemens, Erlangen, Germany) equipped with a 12–channel parallel head coil. In addition to a standard series of clinical sequences, diffusion-weighted images (DWIs) were acquired along 64 non-collinear directions at b = 3000 s/mm^2^, with one non-diffusion weighted image. Acquisition parameters were: 60 axial slices, FOV 30 × 30 cm, slice thickness 2.5 mm, matrix 128 × 128, TR/TE 9200/112 ms, iPAT factor 2. A field map was acquired using two 2D gradient-recalled echo images with TE1/TE2 = 4.76/7.22 msec to assist in the correction of geometric distortions. The acquisition time for the diffusion dataset was 9:40 min.

### Diffusion processing

Diffusion MRI data were preprocessed as described previously [26]. Preprocessing methods included correction for head movement with rotation of the b-matrix, detection and removal of signal intensity outliers, and correction for geometric distortions and intensity inhomogeneity. Maps of FA and MD were calculated using MRtrix 0.2.9.

A custom FA template, generated using the scripts provided with the Advanced Normalization Tools Software (ANTS) package (http://picsl.upenn.edu/ANTS/) [27], was derived from all subjects. We used ANTS symmetric diffeomeophic registrations using symmetric image normalization (Greedy SyN). The Johns Hopkins University (JHU) 1 mm FA was used for the initial rigid body registration to generate the template. The JHU atlas [28] was normalized to this study template using symmetric diffeomorphic registration. JHU atlas ROIs were subsequently transformed to the individual datasets in native space by applying the inverse transform.

### Tract-based spatial statistics (TBSS)

Tract-based spatial statistics analysis was performed with the Functional MRI of the Brain (FMRIB) Software Library (FSL) package version 5.0 (www.fmrib.ox.ac.uk/fsl/tbss) [29] which is a fully automated whole brain analysis technique that uses voxel-wise statistics on DTI data while simultaneously minimizing the effects of misalignment [29]. Briefly, the main steps were a) non-linear alignment of FA images to 1x1x1 mm MNI152 standard space, b) creation of the mean FA image and its white matter “skeleton” representing the tracts that are common to all subjects (mean FA skeleton threshold was 0.2), c) projection of individual FA maps onto the image skeleton, d) projection of individual non-FA maps (e.g. MD) using the projections obtained from FA. We performed voxel-wise statistical analysis on the skeleton, with statistical tests as described below.

### Region of interest (ROI) analysis

**Fig. 1:**
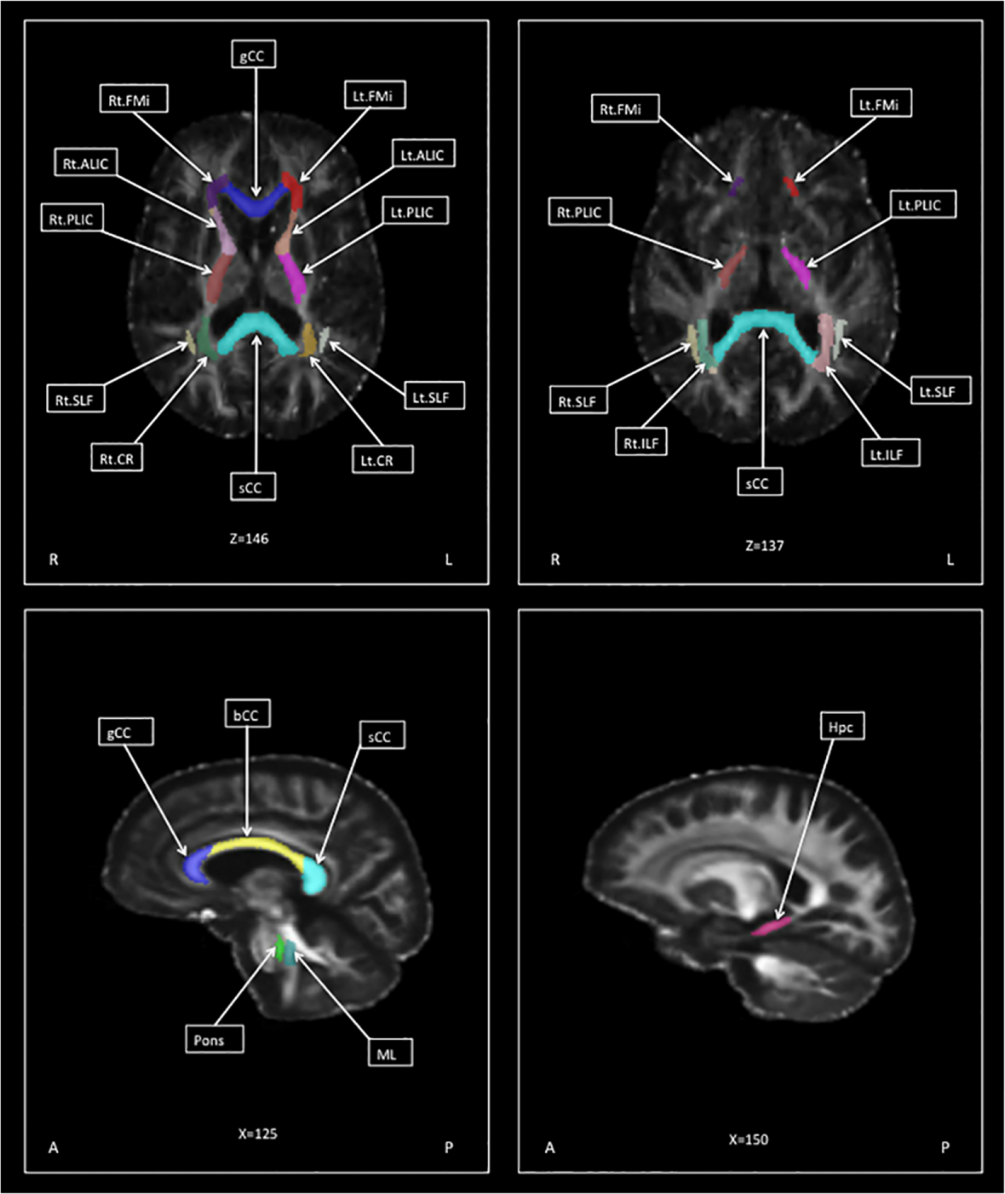
Regions of interest (ROIs) used in the analysis, overlaid on subjects’ template

Using the JHU atlas in subject space, we performed a ROI analysis of the diffusion tensor data in 21 regions; non-midline structures were measured on both sides separately. The regions that were studied are listed in Table 1. Using ITK-Snap software, placement of ROIs was confirmed by one rater (A.R.A). Mean FA and MD values were extracted for each region as shown in Fig. 1.

**Table 1:** List of regions-of-interest investigated in this study

ROIs	
Corticospinal tract	
Corona radiata (CR) (right and left) Medial lemniscus (ML) (right and left) Pons (midline) Posterior internal capsule (PLIC) (right and left)	
Callosal tracts	
Forceps minor FMi) (right and left) Genu corpus callosum (gCC) (midline) Body corpus callosum (bCC) (midline) Splenium corpus callosum (sCC) (midline)	
Association fibers	
Superior longitudinal fasciculus (SLF) (right and left) Inferior longitudinal fasciculus (ILF) (right and left)	
Other extramotor tracts	
Cinglum (Cg) (midline) Hippocampus (Hpc) (right and left) Anterior limb of internal capsule (ALIC) (right and left)

### Statistical analysis

Statistical analyses for ROI measurements were performed using Statistical Package for the Social Sciences (SPSS) for Mac (ver. 23.0, SPSS Inc., Chicago, IL, USA). Mean and standard deviation values were calculated for each variable. All data were tested for normality using the Shapiro-Wilk test. For data that were normally distributed, we used the paired-sample t-test. Significant results were set at the level of *p* < 0.05. For data that were not normally distributed, group differences were analyzed by Wilcoxon rank test, with a threshold for significance of p < 0.05. To confirm our results, MANOVA was performed on the significant results correcting for age and gender as covariant.

The selected ROI were used to explore the relationship between FA and MD with disease severity (using ALSRFRS-R) [22] and disease duration from the time of onset of symptoms until the time of the test for each patient over time using a Pearson correlation. The ALSFRS-R is a scale with 12 domains, with a maximum score of 48 which indicates a lack of symptoms and disability, and a lower score indicates worse disability.

TBSS statistical analysis used a paired t-test design to detect changes between two time-points from the same group of patients. To correct for multiple comparisons across space, we employed permutation testing (5000 permutations) and threshold-free cluster enhancement (TFCE; [30]). We consider results to be significant at a fully corrected *p* < 0.05.

## Results

### Subjects

Twenty-three patients with ALS were studied (16 males, 7 females, mean age 59 years, mean ALSFRS-R score of 39, mean duration at first scan of 27.6 months). These subjects were part of a cohort of 30 ALS patients previously reported [6]. Of the original 30 subjects, 6 declined to have a second scan, usually because of increasing difficulty with mobility and one was deceased before the second scan. Of these 7 patients who did not return for follow-up examination there were 4 males and 3 females. The mean age was 66 years, the mean ALS FRSR score was 36.7 and the mean duration was 16.8 months. Of the 23 participants, 10 had cognitive impairment (ACE-III score of < 88) and one had abnormal frontal lobe features (FAB< 12).

The clinical details of the participants are summarized in Table 2. The clinical features of the individual ALS subjects are shown in Table 3. The timing of the first scan ranged from 3 to 112 months after the date of onset of ALS (mean 27.6 months). Three patients had long disease duration (patient 4: 65 months, patient 7: 68 months; and patient 16: 112 months). In the interval between the first and second scans there was a small but significant decline in the ALSFRS-R (*p* = 0.032).

**Table 2:** Summary of clinical data

	At first scan	At second scan	*p* value
Disease duration in months (mean ± SD)	27.6 ± 24	33.0 ± 24	–
ALSFRS–R (Mean ± SD)	39 ± 5	38 ± 4	0.03

**Table 3:** Clinical features of ALS patients

Subjects	Age	Gender	Handedness	El Escorial category	Phenotype^a^	Site of Onset^b^	Riluzole	Disease duration (months)^c^
1	57	Male	Right	Definite	classic	RLL	Y	15
2	68	Male	Right	Definite	classic	LUL	N	27
3	74	Male	Right	Definite	atypical	RUL	N	48
4	63	Female	Left	Definite	classic	LLL	–	65
5	41	Male	Right	Definite	classic	RUL	Y	22
6	65	Female	Right	Definite	classic	LUL	N	17
7	51	Male	Right	Definite	slow progression	LL (Bilateral)	N	68
8	47	Male	Mixed	Definite	flail	LLL	Y	30
9	57	Male	Right	Definite	flail	LUL	Y	28
10	71	Male	Right	Definite	flail	RLL	Y	19
11	28	Female	Right	Definite	UMN	LUL	Y	13
12	54	Female	Right	Definite	slow progression	LUL	N	2.8
13	66	Female	Right	Definite	classic	RLL	Y	6
14	63	Male	Right	Definite	classic	RUL	N	8
15	60	Female	Right	Definite	classic	LLL	–	31
16	71	Male	Right	Definite	classic	RUL	N	112
17	52	Male	Right	Definite	atypical	LUL	Y	11
18	68	Male	Right	Definite	slow progression	LLL	N	14
19	60	Male	Right	Definite	classic	Bulbar	N	26
20	53	Male	Right	Definite	UMN	LL	Y	27
21	59	Male	Left	Definite	classic	RLL	N	11
22	60	Female	Left	Definite	classic	LLL	N	24
23	71	Male	Right	Definite	classic	Bulbar	N	16

^a^Phenotype as described by Chio et al. 2011 [21]. Those patients with long survival hasve been designated as atypical

^b^RUL: right upper limb, RLL: right lower limb, LUL: left upper limb, LLL: left lower limb, LL: lower limb

^c^disease duration is taken from the time of onset of symptoms until the time of the first scan

### TBSS

Whole brain analysis performed using TBSS showed no significant difference between the first and second scans for ALS subjects. ROI analyses were then used to measure changes in specific brain regions.

### ROI studies

For the motor pathways, there was only a small change in FA between the two scans. In some patients there was a decrease, and in other patients there was an increase in FA. Table 4 shows the mean FA for the first and second scans. There were only small differences between the mean values. Significant increases (*p* < 0.05, uncorrected) were observed in the FA along the cortico-spinal tract at the right medial lemniscus (ML), pons, white matter tracts of the right hippocampus and the right anterior limb of internal capsule (ALIC). However, after correcting for multiple comparisons these differences would not be significant. Using MD, there were no significant changes between ROIs over time in any level of the motor pathways (data not shown).

**Table 4:** Comparison of FA at first and second scans

ROI	FA Mean (SD) First scan	FA Mean (SD) Second scan	*p*-value
Right FMi	0.3639 (0.03)	0.3647 (0.03)	0.586
Left FMi	0.3605 (0.03)	0.3618 (0.03)	0.266
gCC	0.4887 (0.04)	0.4908 (0.04)	0.190
bCC	0.5221 (0.04)	0.5230 (0.04)	0.689
sCC	0.5884 (0.03)	0.5909 (0.03)	0.124
Right CR	0.4076 (0.03)	0.4076 (0.03)	0.988
Left CR	0.4129 (0.03)	0.4133 (0.03)	0.801
Right PLIC	0.5517 (0.03)	0.5525 (0.03)	0.700
Left PLIC	0.5493 (0.03)	0.5520 (0.03)	0.220
Right ML	0.4912 (0.02)	0.4988 (0.02)	0.029
Left ML	0.4958 (0.02)	0.5007 (0.03)	0.163
Pons	0.3879 (0.03)	0.3956 (0.03)	0.032
Right ILF	0.4893 (0.03)	0.4895 (0.03)	0.306
Left ILF	0.5053 (0.03)	0.5031 (0.04)	0.907
Right SLF	0.4293 (0.02)	0.4283 (0.03)	0.506
Left SLF	0.4216 (0.03)	0.4218 (0.03)	0.898
Right ALIC	0.4803 (0.03)	0.4846 (0.03)	0.040
Left ALIC	0.4732 (0.03)	0.4769 (0.03)	0.169
Right Hpc	0.3339 (0.02)	0.3402 (0.03)	0.038
Left Hpc	0.3364 (0.03)	0.3401 (0.02)	0.243
Right Cg	0.4042 (0.03)	0.4046 (0.03)	0.832
Left Cg	0.4171 (0.03)	0.4192 (0.03)	0.177

### Correlation of FA with ALSFRS-R and disease duration

We investigated the correlation of FA with ALSFRS-R and disease duration at both time points, as shown in Table 5. At the first scan, the only significant findings were negative correlations the genu of CC, bilateral forceps minor and bilateral ILF (*p* < 0.05, uncorrected). At the time of the second scan, there were no significant correlations. For disease duration, at the first scan, there was a significant negative correlation was in bilateral ALIC (*p* < 0.05, uncorrected). At second scan, there were significant negative correlations in the right ALIC and bilateral ILF. However, these findings would not be significant after correcting for multiple comparisons.

**Table 5:** Correlation of FA with clinical measures

ROI	ALSFRS-R (Baseline scan)		ALSFRS-R (6– month scan)		Disease duration (Baseline scan)		Disease duration (6– month scan)	
	Pearson Correlation	*p*-value (2–tailed)	Pearson Correlation	*p*-value (2–tailed)	Pearson Correlation	*p*-value (2–tailed)	Pearson Correlation	*p*-value (2–tailed)
Rt.CR	−0.270	0.250	0.287	0.282	− 0.362	0.090	−0.317	0.141
Lt.CR	− 0.236	0.317	0.308	0.246	−0.249	0.253	− 0.195	0.372
Rt.ML	0.103	0.667	0.142	0.600	− 0.120	0.586	0.047	0.830
Lt.ML	0.008	0.972	0.206	0.444	−0.118	0.592	− 0.069	0.755
Rt.PLIC	− 0.178	0.453	− 0.048	0.860	0.069	0.753	−0.179	0.414
Lt.PLIC	−0.127	0.595	−0.141	0.601	−0.041	0.852	−0.255	0.239
Pons	−0.134	0.574	−0.183	0.497	−0.064	0.772	−0.298	0.168
Rt.FMi	−0.649	0.002	−0.173	0.521	−0.354	0.098	−0.378	0.076
Lt.FMi	−0.560	0.010	−0.051	0.851	−0.342	0.110	−0.396	0.061
gCC	−0.542	0.014	0.185	0.493	− 0.203	0.352	−0.180	0.410
bCC	−0.232	0.325	−0.012	0.966	−0.169	0.442	−0.207	0.344
sCC	−0.372	0.106	−0.272	0.308	−0.138	0.530	−0.271	0.211
Rt.ILF	−0.594	0.006	−0.266	0.319	−0.404	0.056	−0.431	0.040
Lt.ILF	−0.597	0.005	− 0.088	0.745	−0.333	0.120	−0.435	0.038
Rt.SLF	−0.399	0.081	0.123	0.650	−0.325	0.130	−0.333	0.120
Lt.SLF	−0.389	0.090	0.271	0.311	−0.245	0.261	−0.315	0.143
Rt.Cg	−0.333	0.152	0.091	0.736	−0.236	0.278	−0.310	0.150
Lt.Cg	−0.373	0.105	−0.063	0.815	−0.196	0.370	−0.178	0.417
Rt.Hpc	0.123	0.605	−0.103	0.704	0.132	0.547	−0.007	0.976
Lt.Hpc	−0.223	0.346	−0.217	0.419	−0.056	0.799	−0.276	0.202
Rt.ALIC	−0.349	0.132	0.130	0.632	−0.596	0.003	−0.535	0.009
Lt.ALIC	−0.381	0.098	0.115	0.671	−0.441	0.035	−0.399	0.059

### Correlation of MD with ALS FRS-R and disease duration

The correlations of FA with ALSFRS-R and with disease duration at both time points are shown in Table 6. At the first scan, there were significant positive correlations between MD and ALSFRS-R scores in the left hippocampus, bilateral ALIC, bilateral ILF and SLF (*p* < 0.05, uncorrected). Bilateral cingulum, forceps minor and the genu of CC also showed significant positive correlation (p < 0.05, uncorrected). In the motor pathways, MD at baseline correlated with ALSFRS-R only in the corona radiata (p < 0.05, uncorrected). At the second scan, the only significant correlations were in the right hippocampus (p < 0.05, uncorrected). However, these would not be significant after correcting for multiple comparisons.

**Table 6:** Correlations of MD with clinical measures

ROI	ALSFRS-R (Baseline)		ALSFRS-R (6– month)		Disease duration (Baseline)		Disease duration (6– month)	
	Pearson Correlation	*p*-value (2–tailed)	Pearson Correlation	*p*-value (2–tailed)	Pearson Correlation	*p*-value (2–tailed)	Pearson Correlation	*p*-value (2–tailed)
Rt.CR	0.468	0.037	0.088	0.745	0.477	0.021	0.455	0.029
Lt.CR	0.493	0.027	−0.008	0.977	0.416	0.048	0.390	0.066
Rt.ML	0.144	0.544	0.241	0.368	0.035	0.873	0.094	0.669
Lt.ML	0.224	0.342	0.202	0.453	0.174	0.428	0.281	0.195
Rt.PLIC	−0.301	.198	−0.195	0.47	−0.277	.201	−.407	.054
Lt.PLIC	−0.133	.575	−0.299	.837	−.252	.247	−.240	.270
pons	0.206	0.384	0.236	0.379	−0.115	0.602	0.130	0.555
Rt.FMi	0.530	0.016	0.212	0.430	0.351	0.101	0.344	0.109
Lt.FMi	0.651	0.0028	0.257	0.337	0.474	0.022	0.474	0.022
gCC	0.508	0.022	0.313	0.238	0.266	0.219	0.285	0.188
bCC	0.313	0.179	−0.034	0.901	0.193	0.378	0.308	0.153
sCC	0.362	0.117	0.276	0.301	0.089	0.686	0.214	0.328
Rt.ILF	0.652	0.002	0.358	0.173	0.445	0.033	0.520	0.011
Lt.ILF	0.621	0.003	0.256	0.338	0.353	0.098	0.438	0.037
Rt.SLF	0.516	0.020	0.223	0.406	0.445	0.033	0.446	0.033
Lt.SLF	0.623	0.003	0.059	0.829	0.497	0.016	0.530	0.009
Rt.Cg	0.469	0.037	0.189	0.482	0.295	0.171	0.307	0.154
Lt.Cg	0.582	0.007	0.232	0.388	0.307	0.154	0.195	0.372
Rt.Hpc	0.310	0.183	0.417	0.108	−0.069	0.754	0.069	0.754
Lt.Hpc	0.478	0.033	0.553	0.026	−0.146	0.507	−0.001	0.995
Rt.ALIC	0.551	0.012	0.310	0.242	0.647	0.001	0.449	0.031
Lt.ALIC	0.702	0.001	0.311	0.241	0.459	0.027	0.602	0.002

For MD, at first scan, the only significant correlations with disease duration were in the ALIC, corona radiata, SLF, right ILF and left forceps minor (*p* < 0.05, uncorrected). At second scan, MD had significant correlation with disease duration in the ALIC, ILF and SLF. Left forceps minor and right corona radiata showed a positive correlation with disease duration (p < 0.05, uncorrected). However, these would not be significant after correction for multiple comparisons.

### Correlation between change in FA and change in ALSFRS-R

The correlations between the changes in FA with the changes in ALSFRS-R in different ROIs is shown in Table 7. Statistically significant correlations were observed only in the splenium of the corpus callosum and the right cingulum (p < 0.05, uncorrected), and this would not be significant after correcting for multiple comparisons.

**Table 7:** Correlation between change in FA and change in ALSFRS-R

ROI	Pearson correlation	*p*-value (2 tailed)
Rt.sCR	−0.009	0.969
Lt.sCR	0.066	0.764
Rt.PLIC	−0.140	0.523
Lt.PLIC	−0.016	0.942
Rt.ML	−0.362	0.090
Lt.ML	−0.186	0.395
Pons	−0.257	0.237
Rt.FMi	−0.150	0.496
Lt.FMi	−0.330	0.124
gCC	−0.095	0.665
bCC	−0.317	0.140
sCC	−0.454	0.029
Rt.ILF	−0.188	0.391
Lt.ILF	−0.364	0.088
Rt.SLF	−0.045	0.840
Lt.SLF	−0.072	0.744
Rt.Cg	−0.534	0.009
Lt.Cg	0.329	0.125
Rt.Hpc	−0.392	0.064
Lt.Hpc	−0.376	0.077
Rt.ALIC	−0.156	0.476
Lt.ALIC	−0.287	0.185

## Discussion

This study was performed to determine the usefulness of DTI of WM tracts in ALS, as a measure of disease progression over a 6–month interval. The ability to measure the progression of ALS using imaging is important for use in prognosis and in clinical trials, and to understand disease pathogenesis [31]. There has been interest in the role of ROI studies in DTI of fiber tracts to evaluate progression of ALS [32, 33] but the results have been variable. A summary of the results of other longitudinal studies of ALS is shown in Table 8. Our study has analyzed the results of FA and MD, which were shown to differ between ALS and controls in our previous study [6].

**Table 8:** Previous serial ROI studies of DTI in ALS

Study	Number of subjects	Mean Age (sd) at first study	Mean ALS FRS-R (sd) at first study	Mean ALS FRS R (sd) at second study	Field Strength	Duration of study	Significant ROI changes
Kwan et al. [8]	9	57.2 (12.6)	40.2 (6.3)	34.1 (9.8)	3 T	Mean of 1.26 years	No change in CR, pons, CST
Steinbach [49]	16	62.1 (11.7)	41.0 (3.6)	38.2 (4.6)	3 T	3 months	No change in CST, Increased connectivity in Hpc
Cardenas–Blanco [54]	34	57.3 (9.9)	40.2 (4.4)	37.9 (5.3)	3 T	Mean of 6 months	No change over time in ALS group
Zhang [36]	17	57.3 (10)	35.1 (7.1)	29.2 (9.3)	4 T	Mean of 8.1 months	Significant decline in FA in R CST
Keil [52]	15	61.5 (10.9)	36.3 (9.0)	na	1.5 T	6 months	Decline in FA in CST
Nickerson [14]	2	48	na	na	3 T	12 months	Decrease in FA in CST
Mitsumoto [17]	30	52.6 (10.9)	36.4 (7.8)	na	1.5 T	9.2 months	No change in CST with FA
Menke [48]	27	61 (11)	35 (6)	na	3 T	Mean 16 months	No change over time
Bede et al. [47]	32	59.9 (9.9)	39.31 (6.4)	33.88 (7.8)	3 T	Mean of 273 days	No change in DTI over time

na = not available

Our study showed that over this time period there was some evidence of clinical progression of ALS patients as seen by a decline in clinical scores of motor function and cognition, but this change was small. Over this time interval there was no significant change in DTI measures using TBSS. ROI analysis of FA and MD revealed some significant changes, however, these would not be significant after correcting for multiple comparisons. There have been inconsistent findings in other serial studies (Table 8), but our work agrees with those who found little change over time.

Using the uncorrected *p* values, there were some minor changes in the motor pathways over 6 months observation. DTI changes in motor pathways over time would be expected in ALS, which involves degeneration of the motor pathways. Previous studies have shown some evidence of progressive decrease in FA in the CST over time [33, 34]. We found changes only in the right hemisphere, which is consistent with previous work by Steinbach et al. [35]. DTI studies using an ROI method showed a bilateral reduction in FA along the CST [34] while other studies found changes in CST to be confined to the right hemisphere [36]. A recent study from our group has found that handedness has an effect on the site of onset and the spread of pathology [37, 38] and that there is asymmetry of atrophy of the motor cortex in ALS [39]. The greater change in the distal portions of the intracranial CST suggests a pattern of distal degeneration. This has also been reported in previous studies [40, 41] and could indicate a dying back of the CST. We found some evidence of progressive changes in the hippocampus. There is known to be atrophy of the hippocampus in ALS [42, 43] and there has been a previous study showing hippocampal abnormality at the advanced stage of ALS [44], but this is the first to show changes over time.

The lack of significant change between scans could be due to the relatively short 6-month interval between scans, as other studies have reported that longer intervals have shown significant changes from baseline [14]. Another reason would be that the patients did not show major change in clinical features, and indeed some of the patients had slow progression. This is a common problem in studies of ALS, where patients with slow progression are often available for inclusion in research projects. Another possibility for the lack of significant change could be the small sample size, and increasing the participant numbers may reveal statistically significant changes. DTI analysis was performed using data that was acquired using optimized parameters for a HARDI analysis, which may affect results slightly.

However, degeneration of upper motor neurons is an early event in ALS [38, 45] and white matter tracts may already be substantially damaged by the time of onset of symptoms. The evidence for early damage to upper motor neurons comes from studies showing early changes in cortical excitability [45] and also our previous study that showed that upper motor neuron signs appear before lower motor neuron signs as the disease spreads [38]. It has been estimated that, because of compensation by collateral sprouting, weakness does occur not until many lower motor neurons are lost, and that clinical signs of ALS follow a long subclinical phase [46]. Other studies have shown little change in DTI over time and the authors have suggested that this is because motor tracts are lost early in the disease. A more recent paper also shows that there is little change in DTI over time, although there is progressive loss of grey matter [35, 47–49].

## Conclusions

In conclusion, there has been previous evidence of substantial DTI changes of WM in ALS, particularly in the CST [50], frontocallosal connections [51] and limbic pathways [52, 53]. However, our study finds little evidence to support using longitudinal DTI studies to follow patients.

## Abbreviations

ACE III: Addenbrookes cognitive examination III
ALS FRSR: ALS functional rating scale revised
ALS: Amyotrophic lateral sclerosis
ANTS: Advance normalization tools software
CST: Corticospinal tract
DTI: Diffusion tensor imaging
FA: Fractional anisotropy
FAB: Frontal assessment battery
FMRIB: Functional MRI of the Brain
FSL: FMRIB software library
GM: Gray matter
HREC: Human research ethics committee
JHU: Johns Hopkins University
MD: Mean diffusivity
MRI: Magnetic resonance imaging
RBWH: Royal Brisbane and Women’s Hospital
ROI: Region of interest
SPSS: Statistical Package for the Social Sciences
TBSS: Tract based spatial statistics
WM: White matter
